# Comparative analysis of Salivette® and paraffin gum preparations for establishment of a metaproteomics analysis pipeline for stimulated human saliva

**DOI:** 10.1080/20002297.2018.1428006

**Published:** 2018-01-24

**Authors:** Alexander Rabe, Manuela Gesell Salazar, Stephan Fuchs, Thomas Kocher, Uwe Völker

**Affiliations:** ^a^ Interfaculty Institute for Genetics and Functional Genomics, Department of Functional Genomics, University Medicine Greifswald, Greifswald, Germany; ^b^ Department of Infectious Diseases, Division of Nosocomial Pathogens and Antibiotic Resistances, Robert Koch-Institute, Wernigerode, Germany; ^c^ Department of Restorative Dentistry, Periodontology, Endodontology, and Preventive and Pediatric Dentistry, University Medicine Greifswald, Greifswald, Germany

**Keywords:** Saliva, metaproteomics, human oral microbiome, whole saliva proteomics, collection method, nLC-MS/MS

## Abstract

The value of saliva as a diagnostic tool can be increased by taxonomic and functional analyses of the microbiota as recently demonstrated. In this proof-of-principle study, we compare two collection methods (Salivette® (SV) and paraffin gum (PG)) for stimulated saliva from five healthy participants and present a workflow including PG preparation which is suitable for metaproteomics.

For a better understanding of the microbial species composition in health and disease of the oral cavity [1,2], saliva offers a wide range of possibilities as shown in metagenomic studies [3–5]. In addition, metaproteomics provides detailed impressions of active metabolic pathways under certain environmental conditions, which cannot be accomplished by metagenomics [6–8]. First metaproteome studies for saliva have already been performed [9–12]. Here, we conducted a comparative proof-of-principle study for two saliva-stimulating collection methods (Salivette® (SV) and paraffin gum (PG)) to identify the most suitable way to perform metaproteome studies on human saliva.

We collected stimulated saliva from five healthy dental students (three men and two women) aged 20–30 years on two consecutive days. Under the supervision of an experienced dentist, the students examined each other and none of them had a probing depth of ≥4 mm. Based on a questionnaire we ensured that all participants met our inclusion criteria (Supplemental Table 1).

All subjects were chewing on a PG for 1 min. Within this minute all volunteers spat saliva into a sterile 50 ml Falcon tube for several times. On the next day, the participants had to chew on the SV for 1 min and the soaked cotton roll was transferred into a specific salivation vessel. Previous experiments showed that the order of the chosen saliva collection methods had no influence on the results (data not shown). Afterwards, all samples were centrifuged for 15 min at 11,500 g (4°C). Saliva collected by PG was separated into supernatant (PG_SN) and pellet (PG_P). SV samples were again centrifuged for 30 min at 17,000 g at 4°C (Salivette supernatant – SV_SN and Salivette pellet – SV_P). For SV_P only a tiny pellet was seen. Pellets were resuspended in 700 μl (PG_P) and 300 μl (SV_P) TE-Buffer. Ultrasound treated pellets were centrifuged for further separation (PG_P_SN, PG_P_P, SV_P_SN) as presented in Figure 1 and Supplemental Table 2. For the SV_P samples no pellet was seen after centrifugation.

**Figure 1.**
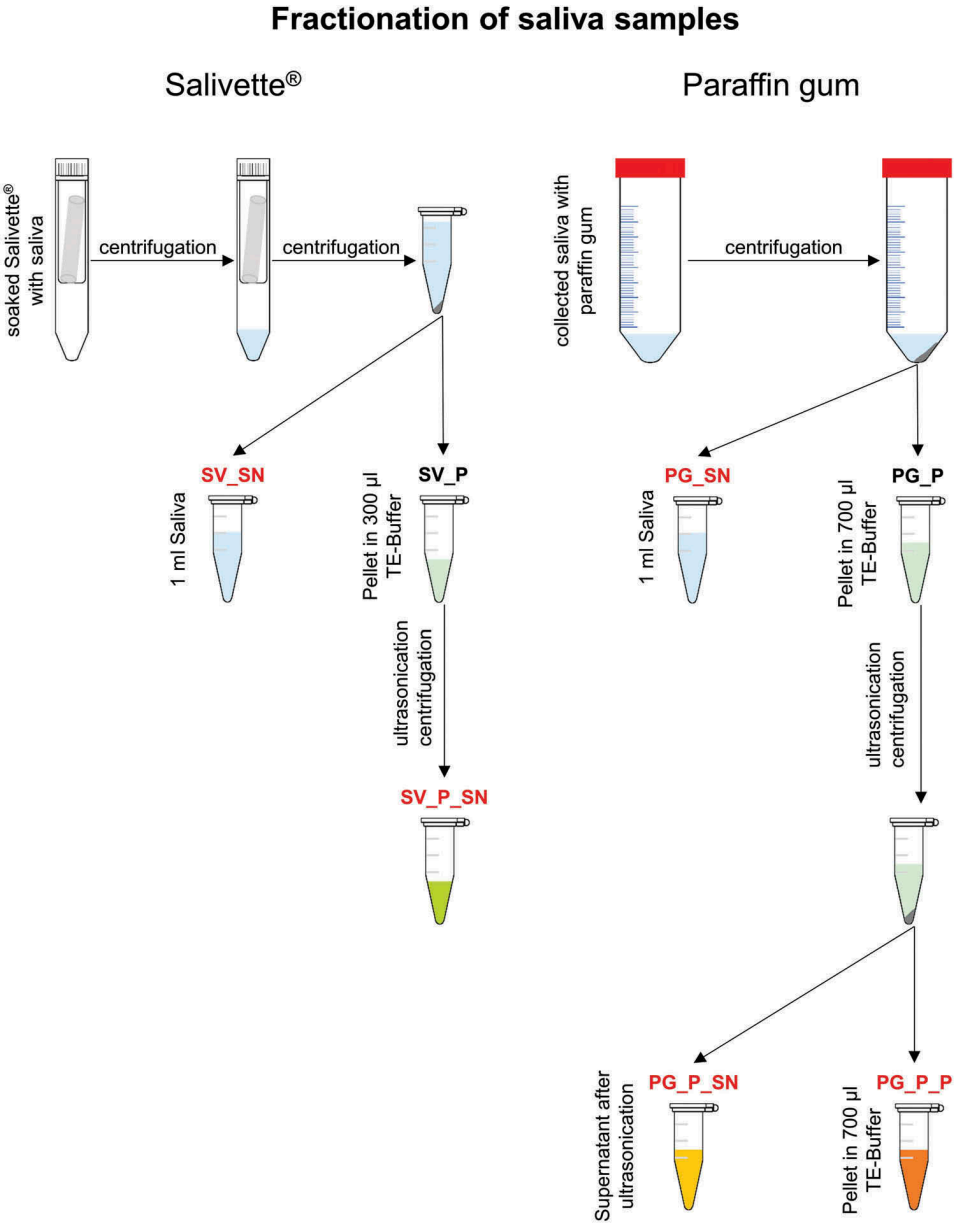
Fractionation procedure of saliva samples collected with Salivette® and paraffin gum. Fractions labeled in red were used for proteome analysis.

Protein precipitation of each fraction (1,000 μl – SV_SN, PG_SN; 700 μl – PG_P_P, PG_P_SN, 300 μl – SV_P_SN) was conducted with TCA. Depending on the size of the resulting pellet, it was dissolved in an 8 M Urea and 2 M Thiourea solution (Supplemental Table 3). Protein concentrations of the lysates were determined using a Bradford Assay (BSA standard curve) [13]. Four micrograms of protein were reduced (dithiothreitol), alkylated (iodo acetamid) and digested with trypsin (ratio 1:25 w/w) for 17 h. Peptide lysates were desalted with two microgram ZipTip-μC18-tips. Tryptic peptide mixtures were analyzed in triplicates by shotgun nano LC MS/MS on an Ultimate® 3000 Nano LC connected to a Q Exactive plus (Supplemental Table 4).

Seventy-five MS-raw files were analyzed as one batch (Supplemental Table 5) with the Proteome Discoverer (v2.0.0.802) software using a database (size: 622 MB) including 20,154 sequences from the *Homo sapiens* proteome (UniProtKB/Swissprot, www.uniprot.org, 01/06/16) [14] and 1,079,644 sequences from 371 different species of the Human Oral Microbiome Database (HOMD, www.homd.org, 12/08/2016) [15,16]. Protein groups were accepted, if covered with ≥ 2peptides and identified in at least two out of three technical replicates. Based on the Lowest-Common-Ancestor-Algorithm-Approach [17] prophane (www.prophane.de, version 2.1.05) was used to perform taxonomic assignment using NCBI [18], BLASTP [19,20] and our database; and functional assignment using COG/KOG [21].

Saliva collection with the PG resulted in a higher volume of saliva (4.1 ± 0.8 ml) compared to the SV (1.9 ± 0.1 ml), which is in accordance with a previous report [22], and yielded also higher protein levels (Supplemental Table 3).

Relative quantification based on NSAF values (normalized spectral abundance factor) revealed that *Homo sapiens* made up the biggest proportion of spectral counts, which differed between the two saliva collection methods and fractions (Supplemental Table 3).

Regarding the human proteome, we refer to the paper by Golatowski et al. [22], which has extensively examined the human proteome data generated by SV and PG preparations. Compared to the previous study, we identified more human proteins, which is expected since we used more advanced instrumentation (QExactive plus vs. Orbitrap Velos). However, an overlap of around 76.0% was reached comparing the same fractions (Supplemental Figure 2) [22].

With regard to bacterial proteins, more than three times more protein groups were identified (Figure 2(a)) using the PG (PG_P_SN: 1,005 protein groups) compared to the SV (SV_P_SN: 313 protein groups). Recent reports identified 1,946 [9] and 2,234 [10] bacterial proteins in human saliva. We assume that our lower protein identification rate is caused by more stringent filter parameters (paragraph 5, Supplemental Table 5) and the use of unique rather than distinct peptides. Furthermore, our study included only five subjects in comparison to other metaproteome studies [9,10].

**Figure 2.**
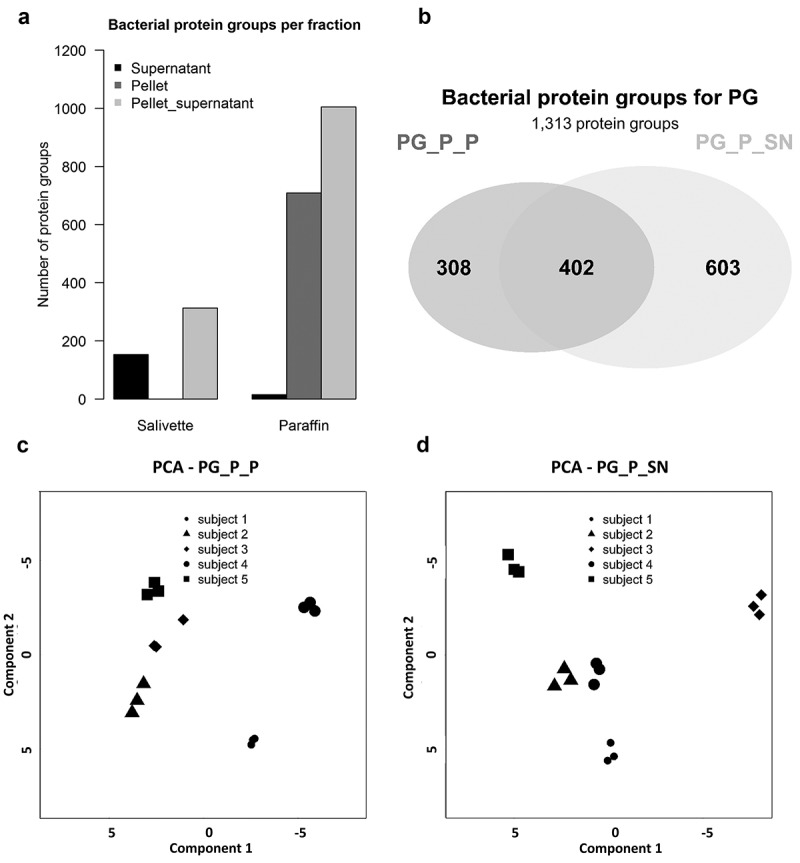
Evaluation of protein identification rate for two stimulated saliva collection methods. (A) The number of identified salivary bacterial proteins for each fraction collected with Salivette® and paraffin gum, respectively. (B) Venn diagram showing the overlap of the number of proteins for the paraffin gum pellet (PG_P_P) and its supernatant (PG_P_SN) fraction and those which were exclusively identified in one of the two fractions. (C, D) Principal component analysis illustrates the technical reproducibility and biological variability for the paraffin gum pellet and its supernatant fraction based on three technical replicates for each fraction.

A comparison of the two fractions (Figure 2(b)) with the highest numbers of protein groups (PG_P_P and PG_P_SN) revealed that 76.5% of the total of 1,313 protein groups were identified with the PG_P_SN fraction (overlap PG-SV: Supplemental Figure 1). A principal component analysis showed that the inter-subject variability was by far larger (PG_P_P: 31%; PG_P_SN: 31.6%) than the technical variance of the analysis (Figure 2(c,d)) and that the technical variance for the PG_P_P (14.9%) fraction was higher in comparison to the PG_P_SN fraction (13.4%). This technical variance is in accordance with a previous study [22]. The results imply that the PG_P_SN fraction is to be favoured due to the highest protein identification of all fractions and its technical reproducibility.

In total, 38 genera and 90 species could be identified, comparing those fractions of the PG (PG_P_SN) and the SV_P_SN with the highest protein group identification (Figure 3(a,b)). Within both fractions (Figure 3(a) – orange) 25 genera and 37 species were covered including the most prominent genera, like *Actinomyces, Prevotella, Streptococcus* or *Rothia* as in previous analyses [9,10,12]. Thirteen genera like *Granulicatella* and 44 species were exclusively found within the PG_P_SN fraction (green). The SV_P_SN fractions (Figure 3(a) – red) did not provide any new genera but nine species. Since the SV_P_SN fraction does not offer any added value with respect to taxonomy, we suggest using the PG_P_SN fraction.

**Figure 3.**
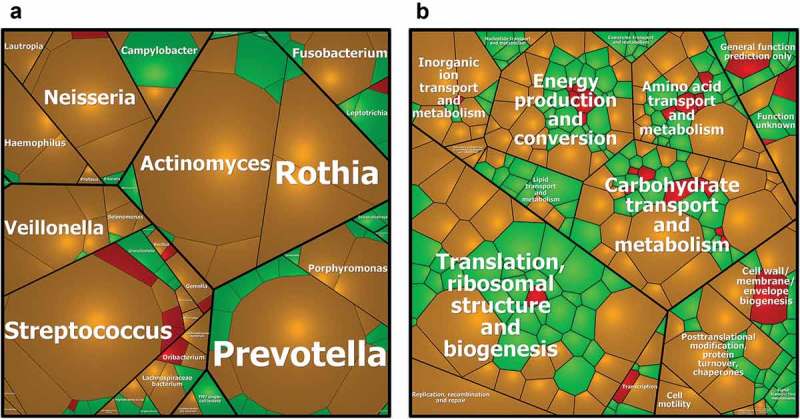
Voronoi treemaps demonstrate taxonomical (A) and functional (B) coverage for SV_P_SN and PG_P_SN. Relative quantification is based on averaged normalized NSAF – values (normalized spectral abundance factor) and presented as polygonal areas. Taxa and protein functions, which were exclusively found in the Salivette® samples (SV_P_SN, red) and paraffin gum samples (PG_P_SN, green) or were identified with both collection methods (orange) are displayed. The treemaps are taxonomically resolved to the species level or functionally to the specific protein function. To keep the figures as brief and clear as possible only the names down to the genus level and to general cellular processes are shown.

Similar observations could also be made on the functional level based on the COG-system (Figure 3(b)) [31]. From 291 COGs found in total, 165 COGs were identified exclusively for the PG_P_SN fraction (Figure 3(b) – green). The main functions (metabolism, cellular processes/signalling and information storage/processing) were covered with both methods (103 COGs – Figure 3(b) – orange). Just a small number of COGs could be observed in the SV_P_SN fractions (23 COGs – Figure 3(b) – red).

Based on this proof-of-principle study, collection of human saliva with the PG turned out as the method of choice for stimulated salivary metaproteomics, because it offers the best results in terms of protein identification, technical reproducibility, taxonomy and functional identification. Future studies must explore larger cohorts to describe the healthy and diseased saliva microbiome.

## Supplementary Material

supplemental_data.zip
