# Supplementary material for: Comparative analysis of Salivette® and paraffin gum preparations for establishment of a metaproteomics analysis pipeline for stimulated human saliva
Source: J Oral Microbiol. 2018 Jan 24;10(1):1428006. doi: 10.1080/20002297.2018.1428006 (PMC5795648; doi:10.1080/20002297.2018.1428006)
Supplement: supplemental_data.zip [file ZJOM_A_1428006_SM6948.zip › supplemental data/comp_pg_sv_JoOM_supplemental.docx]

**Supplemental Table 1:** List of inclusion criteria for subject selection

| ***Inclusion criteria*** |
| --- |
| Female and male subjects (20 - 30 years of age) |
| Present written consent |
| Willingness and ability to meet the requirements of the study |
| No systemic diseases |
| No antibiotics within the last 6 months |
| No drugs, which could influence the PMN- and/or saliva gland function and composition of saliva and sulcus fluid |
| No periodontal / caries therapy within the last 6 months |
| No pregnancy and lactation |
| No simultaneous participation in another clinical trial |
| No smoker |
| No alcohol or drug addicts |

**Supplemental Table 2:** Overview of the fractions of stimulated saliva collected by paraffin gum and Salivette^®^.

| ***fraction*** | ***explanation of abbreviations*** |
| --- | --- |
| ***SV_SN*** | Salivette® - supernatant |
| ***SV_P*** | Salivette® - pellet |
| ***SV_P_SN*** | Salivette® - supernatant after ultrasonication |
| ***PG_SN*** | paraffin gum - supernatant |
| ***PG_P*** | paraffin gum - pellet |
| ***PG_P_P*** | paraffin gum - pellet after ultrasonication |
| ***PG_P_SN*** | paraffin gum - supernatant after ultrasonication |

**Supplemental Table 3:** Overview of general key figures for the individual processed fractions.

|  | ***SV_SN*** | ***SV_P_SN*** | ***PG_SN*** | ***PG_P_P*** | ***PG_P_SN*** |
| --- | --- | --- | --- | --- | --- |
| ***Urea/Thiourea [µl]*** | 150 | 30 | 150 | 500 | 50 |
| ***C_protein_ [µg/µl]*** | 8.2 ± 2.3 | -* | 7.6 ± 0.5 | 13.9 ± 7.1 | 3.75 ± 1.2 |
| ***Protein quantities [mg]*** | 1.4 ± 0.3 | - | 1.1 ± 0.1 | 1.9 ± 0.5 | 0.7 ± 0.3 |
| ***Human spectra [%]*** | 98.1 | 96.9 | 99.1 | 93.2 | 93.5 |
| ***Bacterial spectra [%]*** | 1.9 | 3.1 | 0.9 | 6.8 | 6.5 |

* Protein concentration was below detection limit of the Bradford Assay

**Supplemental Table 4:** Measurement parameters for mass spectrometry

| ***reversed phase liquid chromatography (RPLC)*** | |
| --- | --- |
| instrument | Ultimate 3000 RSLC (Thermo Scientific) |
| trap column | 75 μm inner diameter, packed with 3 μm C18 particles (Acclaim PepMap100, Thermo Scientific) |
| analytical column | Accucore 150-C18, (Thermo Fisher Scientific)  25 cm x 75 μm, 2,6 μm C18 particles, 150 Å pore size |
| buffer system | binary buffer system consisting of 0.1% acetic acid water (buffer A) and 100% ACN in 0.1% acetic acid (buffer B) |
| flow rate | 300 nl/min |
| gradient | linear gradient of buffer B from 2% up to 25% |
| gradient duration | 120 min |
| column oven temperature | 40°C |
| ***mass spectrometry (MS)*** | |
| instrument | Q Exactive plus mass spectrometer (Thermo Scientific) |
| operation mode | data-dependent |
| ***Full MS*** |  |
| MS scan resolution | 70,000 |
| AGC target | 3e6 |
| maximum ion injection time for the MS scan | 120 ms |
| Scan range | 300 to 1650 m/z |
| Spectra data type | profile |
| ***dd-MS2*** |  |
| Resolution | 17,500 |
| MS/MS AGC target | 2e5 |
| maximum ion injection time for the MS/MS scans | 120 ms |
| Spectra data type | centroid |
| selection for MS/MS | 10 most abundant isotope patterns with charge ≥2 from the survey scan |
| isolation window | 3 *m/z* |
| Fixed first mass | 100 m/z |
| dissociation mode | higher energy collisional dissociation (HCD) |
| normalized collision energy | 27.5% |
| dynamic exclusion | 30 s |
| Charge exclusion | 1,>6 |

**Supplemental Table 5:** Search parameters of the Proteome Discoverer software (Thermo Fisher Scientific, v2.0.0.802) for each workflow node.

| ***Workflow node*** | ***Search Parameters Proteome Discoverer*** | |
| --- | --- | --- |
| ***Event dectector*** | mass precision | 2 ppm |
| ***Spectrum selector*** | precursor mass | 350 - 5000 Da |
|  | collision energy | 0 - 1000 |
|  | scan type | full |
| ***SequestHT*** | max. missed cleavage site | 2 |
|  | peptide length | 4 - 144 |
|  | precursor mass tolerance | 10 ppm |
|  | fragment mass tolerance | 0.05 Da |
|  | dynamic modification | Oxidation [+15.995 Da] (M) |
|  | static modification | Carbamidomehtyl [+57.021 Da] (C) |
| ***Percolator*** | target FDR (strict) | 0.01 |
|  | target FDR (relaxed) | 0.05 |
|  | validation based on | q-value |
